# An efficacy trial of brief lifestyle intervention delivered by generalist community nurses (CN SNAP trial)

**DOI:** 10.1186/1472-6955-9-4

**Published:** 2010-02-23

**Authors:** Rachel A Laws, Bibiana C Chan, Anna M Williams, Gawaine Powell Davies, Upali W Jayasinghe, Mahnaz Fanaian, Mark F Harris, the CN SNAP Project Team

**Affiliations:** 1 Centre for Primary Health Care and Equity, School of Public Health and Community Medicine, University of New South Wales, Sydney NSW 2052, Australia

## Abstract

**Background:**

Lifestyle risk factors, in particular smoking, nutrition, alcohol consumption and physical inactivity (SNAP) are the main behavioural risk factors for chronic disease. Primary health care (PHC) has been shown to be an effective setting to address lifestyle risk factors at the individual level. However much of the focus of research to date has been in general practice. Relatively little attention has been paid to the role of nurses working in the PHC setting. Community health nurses are well placed to provide lifestyle intervention as they often see clients in their own homes over an extended period of time, providing the opportunity to offer intervention and enhance motivation through repeated contacts. The overall aim of this study is to evaluate the impact of a brief lifestyle intervention delivered by community nurses in routine practice on changes in clients' SNAP risk factors.

**Methods/Design:**

The trial uses a quasi-experimental design involving four generalist community nursing services in NSW Australia. Services have been randomly allocated to an 'early intervention' group or 'late intervention' (comparison) group. 'Early intervention' sites are provided with training and support for nurses in identifying and offering brief lifestyle intervention for clients during routine consultations. 'Late intervention site' provide usual care and will be offered the study intervention following the final data collection point. A total of 720 generalist community nursing clients will be recruited at the time of referral from participating sites. Data collection consists of 1) telephone surveys with clients at baseline, three months and six months to examine change in SNAP risk factors and readiness to change 2) nurse survey at baseline, six and 12 months to examine changes in nurse confidence, attitudes and practices in the assessment and management of SNAP risk factors 3) semi-structured interviews/focus with nurses, managers and clients in 'early intervention' sites to explore the feasibility, acceptability and sustainability of the intervention.

**Discussion:**

The study will provide evidence about the effectiveness and feasibility of brief lifestyle interventions delivered by generalist community nurses as part of routine practice. This will inform future community nursing practice and PHC policy.

**Trial Registration:**

ACTRN12609001081202

## Background

Smoking, nutrition, alcohol consumption and physical activity (SNAP) are the main lifestyle risk factors for chronic disease and a major cause of morbidity, mortality and impaired functioning [1,2]. In Australia, over 90% of adults do not consume the recommended five serves of vegetables per day, over half do not consume adequate amounts of fruit and 62% are overweight or obese [3]. Approximately one third of adults are classified as physically inactive, one in five (20%) smoke and of the 59% of the population who drink alcohol, 21% do so at a level which would pose a risk to their health [3]. The four SNAP risk factors have been shown to predict a fourfold difference in mortality, equivalent to 14 years in chronological age [4]. In terms of morbidity, the World Health Organization estimates that 80% of cardiovascular disease, 90% of type 2 diabetes and 30% of all cancers could be prevented if lifestyle risk factors were eliminated [1].

Primary health care (PHC) has been identified as a suitable setting to provide individual intervention for lifestyle risk factors because of the accessibility, continuity, and comprehensiveness of the care provided [5]. Evidence also suggests that brief lifestyle interventions delivered in PHC are effective for smoking cessation [6] and 'at-risk alcohol' consumption [7], while moderate to high intensity interventions have shown promise for improving weight, diet and physical activity levels in high risk patients in PHC [8-12].

Within the context of PHC, general practitioners have been the main targets to deliver lifestyle interventions to patients, but relatively little attention has been paid to the role of nurses working in the PHC setting, in particular community health nurses. In Australia, generalist community nurses (GCN) are uniquely placed to provide individual lifestyle intervention because they: 1) frequently see patient with existing chronic conditions that may benefit from lifestyle change; 2) often have ongoing contact with patients over an extended period of time, providing the opportunity to offer intervention and foster motivation through repeated contacts; 3) mainly see clients in their own home providing opportunity for observation of the living situation and intervention with the wider family/carers; 4) may reach disadvantaged individuals and other segments of the population who have poor contact with GPs. Furthermore, our previous research has shown that community health nurses consider the provision of lifestyle intervention an appropriate component of their role as it fits well with their philosophy of providing holistic care, is often relevant to the clients presenting issue and is well accepted by clients [13].

While the health education and promotion role of community nurses is well recognised [13-17], few studies have evaluated the effectiveness of lifestyle interventions provided by community nurses in routine practice. A study in the USA reported that patients who received smoking cessation counselling from a home health care nurse were twice as likely to be continuously abstinent at 12 months compared to those receiving usual nursing care [18]. Community nursing follow up has also been shown to be effective in promoting abstinence and retention rates in outpatient treatment of alcohol dependent patients [19]. While these findings are promising, we are unaware of previous studies that have examined the effectiveness of community nurses delivering intervention across all four lifestyle risk factors in routine practice.

### Study Aim, Objectives and Hypotheses

The overall aim of this study is to evaluate the impact of a brief lifestyle intervention delivered by GCNs in routine practice on changes in clients' SNAP risk factors.

The study has three main objectives:

1. To develop an intervention to facilitate GCNs to undertake lifestyle screening and brief intervention during routine consultations and to promote referral of 'at-risk' clients to available private and public services;

2. To evaluate the impact of these interventions on change in clients' lifestyle risk factors and related mediators (eg stages of change);

3. To describe the indicative costs and resources utilised by the community nurse services in delivering the intervention.

It is hypothesised that:

1. 20% more clients who are at high risk (defined as having at least one lifestyle risk factor) in the intervention group will be offered evidence-based interventions to modify their risk factors compared with clients in the comparison group.

2. Clients in the intervention group will be significantly more likely to progress in their stage of change compared to comparison clients.

3. Clients in the intervention group will have a reduction in their lifestyle risk factor scores compared to clients in the comparison group.

## Methods/Design

### Study Design and Setting

This quasi-experimental study is being conducted in GCN services in the state of NSW, Australia. Within Australia, GCNs can be either registered or enrolled nurses who perform a diversity of roles depending on the service in which they work. The role has traditionally focused on providing a range of nursing care in people's homes such as assisting with activities of daily living, wound management, chronic disease care, continence management, palliative care, medication management, disability and dementia care. Coordination of care with other service providers and health promotion has also traditionally been a major part of their role.

A quasi-experimental design was chosen because it was not feasible to randomise the intervention according to individual patient or practitioners within the services. GCN services have been randomly allocated to an 'early intervention' group or 'late intervention' (comparison) group. 'Early intervention' services are provided with training and support for nurses in identifying and offering brief SNAP intervention for clients during routine consultations (see service-level intervention). Data is being collected from clients in both groups at baseline, three months and six months to examine short and medium term change in SNAP risk factors. This will enable a comparison of client outcomes between early and late intervention groups. After the six month data collection point the 'late intervention' group will be offered the same training and support as the early intervention group.

### Power and Sample Size Calculation

The sample size calculation is based on the primary outcomes of change in continuous self reported measures of lifestyle risk factors rather than differences in proportion of individuals in each risk category (secondary outcomes). There is evidence that risk associated with diet, physical activity and weight is continuous [20-22]. Achieving a shift in risk categories is more difficult to demonstrate in the context of a brief intervention and would require a very large sample size which is beyond the scope and resources of this trial. Hence the focus is on change in continuous measures of lifestyle risk factors as the primary outcomes.

A sample size of 360 clients in each group (total n = 720) is required to have 80% of power to detect the following difference between intervention and comparison groups:

▪ Mean difference of 1 serve of fruit and vegetables consumed (equivalent to 22% increase in the 'early intervention' group);

▪ Mean difference of 1.2 for physical activity scores as measured by a validated brief physical activity assessment tool [23] (equivalent to a 29% increase in mean scores in the 'early intervention' group);

▪ Mean difference of 0.6 for alcohol risk scores as measured by the AUDIT-C tool [24] (equivalent to a 10% reduction in mean scores in the 'early intervention' group);

▪ Mean difference of 4.1 kg in self reported weight (equivalent to a 4.4% weight reduction in the 'early intervention' group).

This is based on 5% significance level and 80% power (β = 0.8 and α = 0.05) allowing 20% lost to follow-up and a design effect of 1.8 due to clustering. The expected differences between intervention and comparison groups has been estimated based on previous research [25]. These differences are also considered meaningful from a population health perspective. Baseline prevalence of risk factors is based on NSW Health Survey 2006 for individuals aged 45-74 years [26].

### Recruitment

#### Generalist Community Nursing Services

Four GCN services were recruited to participate in the trial. An expression of interest to participate in the trial was sent out to all seven Area Health Services(AHS) in NSW. In NSW, AHS are responsible for providing all hospital-and community-based health care apart from general practice and PHC services for specific population groups such as Aboriginal and Torres Strait Islanders. AHS were asked to nominate suitable GCN services which were ranked and selected according to the selection criteria provided in Table 1.

**Table 1:** Selection criteria for recruiting generalist community nursing services to participate in the trial

Types	Selection Criteria
**Inclusion Criteria**	The team deliver GCN services to clients between 30 and 80 years of age The team is able to recruit sufficient numbers of eligible clients into the study in a given recruitment period (based on trial sample size calculation). The individual clinicians are interested in participating and commit to undertaking training and intervention Willingness of the service to accommodate a recruitment officer to work on site to identify and contact potentially eligible clients from client referrals. Willingness of the service to review and modify service assessment protocols to include standard tools for identification SNAP risk factors (if not already included). The team/services has the capacity to participate in SNAP training The team/service has the capacity to deliver SNAP intervention to a given number of clients in a given intervention period. The team/service is broadly representative of community nursing services statewide (choosing 'typical service(s)'). Willingness of the service to participate in project management meetings with study management team body to support the trial implementation (eg service manager and AHS project/recruitment officer).

**Exclusion Criteria:**	Teams/services undergoing concurrent changes in management, structure or service deliver that would impact on capacity to be involved in the trial. Involvement in other service development activities that may contaminant trial activities (eg concurrently delivering self management program for clients).

#### Recruitment of Clients

In order to reach the required sample size, GCN services are required to recruit 180 clients each. Clients referred to participating GCN services meeting the selection criteria (Table 2) are invited to participate in the study. Potential participants are contacted by phone on the day of referral (where ever possible) by a trained local recruitment officer. Clients who consent to take part in the study then participate in a telephone interview to collect baseline data (see data collection). A total of 720 clients (average 180 clients per service) will be recruited at baseline. We expect a 20% loss to follow-up of clients of over the study period, leaving 576 clients (or 288 in each group).

**Table 2:** Selection criteria for recruiting clients to participate in the trial

Types	Selection criteria
**Inclusion criteria**	Clients referred to community nursing services Age 30-80 years Able to read and understand English at a level that enables the client to participate in a telephone administered survey and to understand the participant information sheet.

**Exclusion criteria**	Palliative care clients Clients receiving one off visit or service Clients with significant cognitive impairment (unable to complete telephone administer survey). Clients who are currently receiving help in changing their lifestyle from a health professional (other than their GP) such as a dietitian or exercise physiologist Clients who are currently attending a chronic disease management program such as cardiac rehabilitation, diabetes education program etc. Clients who have attended the generalist community nursing service in the previous six months (and therefore may have already received lifestyle intervention)

### Randomisation and Blinding

Services were randomly allocated to an 'early intervention' or 'late intervention' (comparison) group. Randomisations were performed by a person independent of the research team. Staff involved in the client data collection are independent of those involved in the intervention and blind to which services were randomised to intervention or comparison groups.

### Intervention

Intervention is being undertaken at two levels 1) Service level intervention involving GCN services and nurses, undertaken by the researchers and 2) Client level intervention undertaken by the participating nurses.

#### Service-Level Intervention

At the service-level, the intervention aims to increase the capacity of the GCN to undertake a brief SNAP intervention with clients. The design of the intervention has been informed by our previous research reporting on a theoretical model of the factors shaping the lifestyle risk factor management practices of non GP PHC providers [27]. The model suggests lifestyle risk factor management practices are shaped by: 1) clinician commitment, in particular beliefs about role congruence, client receptiveness and outcome expectations; and 2) clinician capacity, including self-efficacy, role support (provision of training, decision support tools, client education materials and linkages to referral services) and the extent to which lifestyle risk factor management activities fit with clinicians' way of working.

Specifically, the service-level intervention comprises of the following components:

▪ A one day training program for participating nurses delivered by the research team in conjunction with local providers. The training specifically focus on building clinician knowledge, skills and positive attitudes relevant to the model constructs, with a particular focus on developing behaviour change skills such as motivational interviewing and goal setting (learning objectives detailed in Table 3). An emphasis is placed on experiential learning including the use of role-plays with simulated clients (actors), group discussions and activities, which have been shown to be effective in improving knowledge, skills and confidence related to lifestyle risk factor management [28-31].

▪ The integration of standard screening tools and prompts for SNAP risk factors (see additional file 1) into the service specific assessment process used by the nurses (see additional file 1).

▪ The development and dissemination of a local service referral directory to each GCN team to promote referral of clients for ongoing specialist management or more/ongoing intensive lifestyle intervention (such as the proactive telephone Quitline).

▪ The provision of resources to support the implementation of the intervention including a written guide for nurses, written action plans for use with clients on each SNAP risk factor, tape measures for measuring waist circumference, and pedometers to loan out to clients to encourage self monitoring of incidental physical activity

Consultation has been undertaken with participating sites to ensure that intervention activities are feasible to deliver within routine practice. A nurse from each of the early intervention sites has also be seconded to work with the research team to develop the intervention and to support its implementation at the local level.

**Table 3:** Nurse training program: Learning objectives

	Learning Objectives
**Skills**	*At the end of the training program nurses will be able to:* Create a legitimate opening to discuss lifestyle issues with clients Assess lifestyle risk factors and readiness to change using the provided screening tools Tailor their approach to clients stage of change (including managing client resistance, motivational interviewing and goal setting) Use action plans to negotiate goals for behaviour change with the client

**Knowledge/Awareness**	*At the end of the training program nurses will have a knowledge/understanding of:* How individual clinical intervention for lifestyle risk factors fits within broader framework of policies and programs in this area. Key recommendations/targets for behavioural risk factors Strategies to assist clients in making lifestyle changes Behaviour change principles (stage of change, tailoring of advice, importance of multiple interventions over time, small incremental changes in behaviour) Available referral services and how to refer clients for more intensive intervention Lifestyle action plans and their application

**Attitudes**	*The training program will aim to develop positive nurse attitudes with respect to:* The relevance of lifestyle risk factors to their role The scope to make a difference to individuals, families and population health through lifestyle risk factor management The likely effectiveness of brief intervention and how this fits with a range of other interventions at the community and population level Their role as a facilitator of change for all clients rather than a provider of information only for clients interested or able to change Client acceptability and managing client resistance and lack of motivation/capacity for lifestyle change

#### Client- Level Intervention

The aim of the client level intervention is to assist clients to make positive lifestyle changes by enhancing readiness to change, supporting self management knowledge and skills, and increasing self-efficacy. Specifically, the goals of the intervention are to achieve and maintain lifestyle changes consisted with current Australian recommendations [32]:

▪ Moderate physical activity for at least 30 minutes/day including: walking, jogging, swimming, aerobic, ball games, skiing with circuit-type resistance training if possible, twice a week;

▪ A diet low in saturated fats, sucrose and salt with increased portions of vegetables and fruit per day (up to 7 portions) in order to achieve a diet where the percentage of energy from carbohydrates = 50%, saturated fats <10% (and total fats < 30%, protein 1 g/kg ideal body weight per day, fiber 15 g/1000 kcal).

▪ Weight reduction (if overweight) of ≥ 5 kg or 5% of body weight;

▪ Smoking cessation (if smoker);

▪ Limit alcohol intake (if drinking) to ≤ 2 drinks/day, including 1-2 alcohol free days/week.

**Figure 1:**
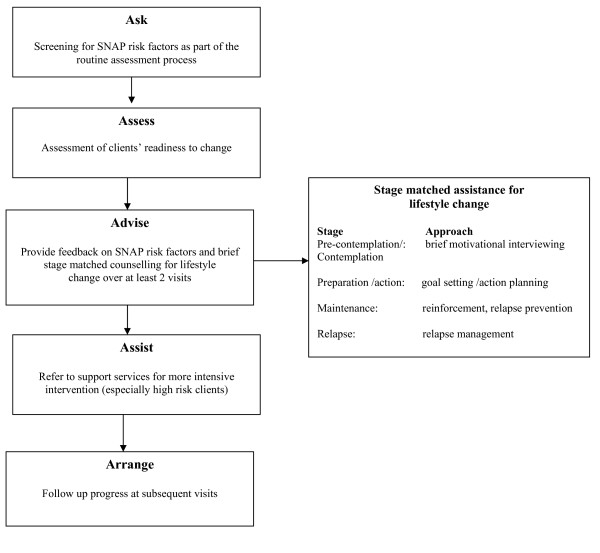
**5As model of brief lifestyle intervention using the transtheoretical model of behaviour change**.

The client intervention involves GCNs providing a brief intervention tailored to a client's readiness to change, in line with the evidenced-based 5As model [5] (Figure 1) for one or more SNAP risk factors. Participating nurses screen clients for lifestyle risk factors as part of the usual assessment process using a screening tool (see additional file 1) incorporated into the service specific assessment forms. A brief lifestyle intervention is then provided over two or more visits and 'at-risk' clients referred to support services (such as proactive quitline) for more intensive intervention. It is intended that the intervention be delivered as part of routine practice in early intervention sites. Nurses are required to use their clinical judgement to determine whether the intervention is appropriate for the client.

The intervention has been informed by the Transtheoretical Model of Behavior Change [33] which postulates that individuals are at different stages of readiness to adopt a new behaviour and that individuals are required to progress through various 'stages of change'. These stages include not thinking about change (pre-contemplation), considering change in the next six months (contemplation), making steps to prepare for change in the next month (preparation), making changes to behaviour (action) and maintaining behaviour change for six months or more (maintenance) [33]. The model hypothesises that the balance of potential risks and benefits of change (decisional balance) and self efficacy predict movement through these various stages [34]. Furthermore, evidence suggests that providing interventions tailored to an individual's stage of change (stage matched) is more effective than providing the same intervention to all individuals [34-37].

### Late Intervention (comparison) group

Two of the four GCN services have been randomly allocated to the 'late intervention' condition. These services will not receive the study intervention till after the six month data collection point and continue to deliver 'usual care'. This will enable the effectiveness of the service and client level intervention to be assessed in comparison to usual practice.

### Study Outcomes, Measurements and Data Collection

The study outcomes, measurement tools and timeframe for data collection is summarised in Table 4. The primary outcomes focus on measuring continuous self reported measures of lifestyle risk factors, while secondary outcomes focus on the proportion of individuals in 'at-risk' categories. Mediator variables of interest include client's progress in their 'readiness to change' lifestyle behaviours and change in nurse self-reported confidence and attitudes in managing lifestyle risk factors.

**Table 4:** Study Outcomes and Measurement

Outcomes	Measurement	Time point
***Primary Outcomes***		

The number and % of high risk clients offered evidence based interventions to modify their risk factors	Client telephone survey	Baseline, 3 and 6 months

Change in mean physical activity scores as measured by a brief validated physical activity tool [23]	Client telephone survey	Baseline, 3 and 6 months

Change in mean alcohol intake score as measured by the validated AUDIT-C tool [24]	Client telephone survey	Baseline, 3 and 6 months

Change in mean number of serves of fruit and vegetables as measured by validated questions from the NSW Health survey [46]	Client telephone survey	Baseline, 3 and 6 months

Mean self reported weight change	Client telephone survey	Baseline, 3 and 6 months

***Secondary Outcomes***		

Change in the number and percentage of clients who report smoking	Client telephone survey	Baseline, 3 and 6 months

Change in number and percentage of clients reporting adequate levels of physical activity	Client telephone survey	Baseline, 3 and 6 months

Change in the number and percentage of clients reporting 'at risk' alcohol consumption	Client telephone survey	Baseline, 3 and 6 months

Change in the number and percentage of clients consuming >=2 serves of fruit per day	Client telephone survey	Baseline, 3 and 6 months

Change in the number and percentage of clients consuming >=5 serves of vegetables per day	Client telephone survey	Baseline, 3 and 6 months

Change in nurse self reported SNAP risk factor assessment and management scores	Clinician survey	Baseline, 6 and 12 months

***Intermediate outcomes (mediator variables)***		

Progression in stages of change (number and % high clients in each stage of change) as measured by five point intention scales [34]	Client telephone survey	Baseline, 3 and 6 months

Change in nurse self reported confidence and attitude scores for SNAP management measured by nurse survey.	Clinician survey	Baseline, 6 and 12 months

Changes in clients' lifestyle risk factors is self reported and captured through a 15 minute telephone survey conducted with clients at baseline (prior to first nurse visit), three and six months. The telephone survey is being conducted by trained independent data collectors blinded to the group allocation (intervention or comparison) of services. Measures of lifestyle risk factors used in the survey are based on validated tools as detailed in Table 4. Nurse outcomes are being measured using a web-based survey adapted from the Preventive Medicine Attitudes and Activities Questionnaire (PMAAQ) [38] administered at baseline, six and 12 months. All data collection tools have been piloted prior to use.

### Statistical Analysis

#### Client Outcomes

Change in clients' lifestyle risk factors between early intervention and late (comparison) services will be assessed using multilevel models. Because this is a quasi-experimental study, clients in the intervention group are not randomly allocated and may have characteristics that are related to outcomes independent of the intervention [39]. Co-variates will be included in the analysis to adjust for baseline differences between the intervention and control groups [40]. Regression method for clustered data or multilevel models will be used to adjust for confounding client variables such as age, gender, locality (rural versus urban), socio-economic status, existing health conditions and number of contacts with the nurse. Thus, this method is used to estimate intervention effect after adjustment for client characteristics [41].

In the study we will use multilevel models in which three repeated measures (the level 1 units) are nested within clients (level 2 units) [42]. Multilevel models can accommodate unbalanced data due to attrition or missing values. Linear multilevel models would be used for continuous response variables and multilevel logistic models used for binary responses. The independent variables will be intervention (1 = intervention & 0 = control), time (0 = baseline, 1 = three months & 2 = six months) as a continuous variable, sex, age, health status of the client and the interaction between intervention and time. This model will allow us to test for a significant interaction between time and intervention, which would indicate an effect of the intervention on outcomes [40,43].

#### Nurse Outcomes

Change in nurses risk factor management confidence, attitudes and practice scores between early and late intervention services (comparison) will be assessed for clustering and, if this is not significant, comparison will be made between the change in early and late intervention providers using unilevel multivariate methods.

### Qualitative Evaluation

As this is a complex intervention delivered within the context of normal practice, a qualitative evaluation of the implementation process is important in interpreting the study findings [44]. A qualitative evaluation will specifically explore the acceptability, usefulness and sustainability of the study intervention for both GCNs and participating clients.

It is proposed that nurse focus groups be conducted in each early intervention service along with individual interviews with team managers and project officers (n = 8) to explore the:

▪ experience of implementing SNAP risk factor management in routine practice (appropriateness, feasibility, client receptiveness, factors affecting implementation);

▪ usefulness of components of the intervention and additional supports required for sustained uptake;

▪ sustainability of the intervention and issues to consider in transferring to other GCN teams

Semi-structured telephone interviews will also be conducted with a sample of clients (n = 20-25) from early intervention sites who recall receiving lifestyle intervention from a GCN. Clients will be purposefully sampled according to age, gender, health and socio-economic status of clients. The interviews will aim to explore:

▪ acceptability of SNAP screening;

▪ acceptability and usefulness of the brief lifestyle intervention provided by the community nurse;

▪ acceptability and usefulness of referral to support services.

All interview and focus group data would be transcribed verbatim and subject to thematic analysis using Nvivo 8 [45] to identify convergence and divergence of themes.

### Ethics

The project was approved by the UNSW Human Research Ethics Committee (HREC) and the HREC in each AHS. All participants are requested to provide their informed consent.

## Discussion

Lifestyle risk factors need to be tackled at multiple levels including individual intervention provided by health professionals as well as through population health approaches. Much of the focus in health professional interventions to date has been on general practitioners, with relatively little attention paid to nurses outside of general practice. Community health nurses are well placed to deliver lifestyle interventions to clients and their involvement should make these types of intervention more accessible to the population, but little is known about the effectiveness of such interventions delivered in routine practice.

This study is a complex intervention trial examining the effectiveness lifestyle risk factor management delivered by GCNs. The trial is unique in that it is being delivered as part of routine practice (without additional nurse resources) and aims to examine the impact of brief intervention on multiple lifestyle risk factors. The collection of quantitative outcome data will provide new information about the efficacy of such an approach at both the clinician and client level. This will be supplemented by the qualitative evaluation which will provide important contextual information about the feasibility, acceptability and sustainability of this approach for both nurses and clients. Together this information will help inform future community nursing practice and PHC policy including:

• defining the roles of GCNs in assessing and intervening with clients to modify lifestyle risk factors

• identifying competencies for vocational training and continuing education of GCNs

• organisation of care and the place of health promotion interventions in the care pathway for GCN clients

• investment in health promotion programs involving GCNs

• the role of referral pathways linking PHC with support services for lifestyle risk factor management including both public and private providers

The findings and intervention materials of the study will be disseminated using a knowledge transfer exchange strategy. This will include dissemination of key findings using peer reviewed journals, conference presentations, the formulation of simple research summaries tailored for practitioner, service managers and policy makers.

## Competing interests

The authors declare that they have no competing interest in the conduct of this study.

## Authors' contributions

All authors have contributed to study design and have reviewed and approved the final manuscript. In particular, RAL and BC have lead the development of data collection tools and processes, RAL and MFH intervention design and UJW statistical analysis methods. RAL wrote the first draft of the manuscript.

## Pre-publication history

The pre-publication history for this paper can be accessed here:

http://www.biomedcentral.com/1472-6955/9/4/prepub

## Supplementary Material

Additional file 1 **SNAP screening tools and prompts. **This document provides the SNAP screening tools and prompts used in the trial (integrated into the standard nursing assessment forms used by early intervention sites). Click here for file
