# Supplementary material for: An efficacy trial of brief lifestyle intervention delivered by generalist community nurses (CN SNAP trial)
Source: BMC Nurs. 2010 Feb 23;9:4. doi: 10.1186/1472-6955-9-4 (PMC2841173; doi:10.1186/1472-6955-9-4)
Supplement: Additional file 1 — SNAP screening tools and prompts. This document provides the SNAP screening tools and prompts used in the trial (integrated into the standard nursing assessment forms used by early intervention sites). [file 1472-6955-9-4-S1.DOC]

**SNAP Screening Tools and Prompts**

**SMOKING**

1. Smoking Status

Never smoked

Currently smokes *→ assess readiness to quit (Q2)*

Has quit smoking If quit, record when _________

2. Are you interested in trying to quit?

No *→ explore, personalise benefits, leave door open to seek future help*

Unsure *→ Ask about pros and cons of quitting & barriers*

Yes, considering quitting

Yes, attempting to quit now *Assess nicotine dependence & action plan*

*/referral to Quitline 13 7848*

3. Nicotine Dependency (for those ready to quit)

1. How soon after waking up do you smoke your first cigarette? _____

*If first cigarette within 30 minutes of waking probably dependent, discuss nicotine replacement therapy .*

**Action Taken: __________________________________________**

**PHYSICAL ACTIVITY**

- 1. Is there anything stopping you from being physically active (physical impairments/barriers): ___________________________________________________
  2. Would you do a total of 30 minutes of moderate physical activity (such as walking or yard work or any other type of exercise) on most days of the week?

Yes

No *→ assess readiness to become more active*

Not applicable

- 1. Are you interested in doing more physical activity?

No *→ explore, personalise benefits, leave door open to seek future help*

Unsure *→ Ask about pros and cons of becoming more active & barriers*

Yes, considering becoming more active

Yes, attempting to increase activity now *action plan/referral*

**Action Taken: __________________________________________**

**NUTRITION**

| **Nutrition Risk Screening Tool** | | |
| --- | --- | --- |
| **Poor Dietary Intake / Under Nutrition** | | |
| Obvious underweight – frailty? | YES | NO |
| Unintentional weight loss >=5 kg in 1 month? | YES | NO |
| Reduced appetite or reduced food or fluid intake? | YES | NO |
| Mouth or teeth or swallowing problems? | YES | NO |
| Follows a special diet? Type? | YES | NO |
| Unable to shop for food? | YES | NO |
| Unable to prepare food? | YES | NO |
| Unable to feed self? | YES | NO |
| **Overweight/Obesity** (only assess in those 75 years of age or less) | | |
| Obvious overweight (waist circumference in yellow or red range) affecting quality of life?  Waist circumference: ________cm | YES | NO |
| Unintentional weight gain? | YES | NO |
| *If yes to any of the above may be nutritional ‘at risk’ assess readiness to change* | | |
| **Fruit & Vegetables:** | | |
| Do you eat 2 serves of fruit most days? *1 serve is equivalent to a medium size piece of fruit, 2 small pieces of fruit, ½ cup fruit juice* | YES | NO |
| Do you eat 5 serves of vegetables most days? *1 serve is equivalent to ½ cup vegetables, 1 cup salad, 1 medium potato* | YES | NO |
| *If no to any of the above may be nutritionally ‘at risk’ assess readiness to change* | | |

Are you interested in changing your eating habits to improve your health?

No *→ explore, personalise benefits, leave door open to seek future help*

Unsure *→ Ask about pros and cons of dietary change & barriers*

Yes, considering making changes *overweight/obese action plan / referral*

Yes, attempting to make changes now *under nutrition ‘handy hints for nourishing*

*meals’./referral*

**Action Taken: __________________________________________**

**ALCOHOL**

1. Do you drink alcohol?

Never / rarely

2 or less standard drinks on a typical day when drinking

>2 standard drinks on a typical day when drinking

>4 standard drinks on any one occasion

*If > 2 standard drinks a day when drinking ask :*

Number of standard drinks on typical day:____

Frequency: ______

Number of alcohol free days a week ____

*If > 2 standard drinks a day or >4 standard drinks on any one occasion → assess readiness to reduce alcohol intake.*

1. Are you interested in reducing your alcohol intake?

No *→ explore, personalise benefits, leave door open to seek future help*

Unsure *→ Ask about pros and cons of reducing drinking & barriers*

Yes, considering reducing *action plan/referral*

Yes, attempting to reduce now

*Note: Suspect alcohol dependence If drinking >50 standard drinks a week in men or >35 standard drinks a week in women, consider referral to drug & alcohol services*

**Action Taken: __________________________________________**

**Standard drinks:**

1 = can light beer (375ml), middy (285ml) of mid strength beer, small glass wine (100ml), 1 measure spirits (30 ml), 60 ml port/sherry

- 1 can full strength beer = 1.5 units,
- 6 pack of full strength = 9 units, 1 slab/carton = 36 units.
- Average serve of wine (150 ml) or sparkling wine (170 ml) = 1.5 units
- 1 bottle wine = 7.5 units
- 1 can pre-mixed spirits = 1.5, 1 bottle pre-mix spirits = 1.2 units
- 1 bottle of spirits (40% alc) = 22 units
